# The effect of task-irrelevant objects in spatial contextual cueing

**DOI:** 10.3389/fcogn.2024.1336379

**Published:** 2024-03-05

**Authors:** Adrian von Mühlenen, Markus Conci

**Affiliations:** ^1^Department of Psychology, University of Warwick, Coventry, United Kingdom; ^2^Department Psychologie, Ludwig-Maximilians-Universität München, Munich, Germany

**Keywords:** attention, visual search, probability priming, statistical learning, working memory, memory template

## Abstract

During visual search, the spatial configuration of the stimuli can be learned when the same displays are presented repeatedly, thereby guiding attention more efficiently to the target location (contextual cueing effect). This study investigated how the presence of a task-irrelevant object influences the contextual cueing effect. Experiment 1 used a standard T/L search task with “old” display configurations presented repeatedly among “new” displays. A green-filled square appeared at unoccupied locations within the search display. The results showed that the typical contextual cueing effect was strongly reduced when a square was added to the display. In Experiment 2, the contextual cueing effect was reinstated by simply including trials where the square could appear at an occupied location (i.e., underneath the search stimuli). Experiment 3 replicated the previous experiment, showing that the restored contextual cueing effect did not depend on whether the square was actually overlapping with a stimulus or not. The final two experiments introduced a display change in the last epoch. The results showed that the square does not only hinder the acquisition of contextual information but also its manifestation. These findings are discussed in terms of an account where effective contextual learning depends on whether the square is perceived as part of the search display or as part of the display background.

## Introduction

Attentional processes play an important role in how our brain filters and prioritizes incoming sensory information, allowing us to navigate through our rich and sometimes rather complex environment. For instance, salient, rich sensory features in our environment, such as sudden loud noises or a bright, flashing warning light (e.g., Yantis, 1993) might capture our attention in a stimulus-driven manner and thus influence goal-directed behavior. Effective processing of such salient information is essential for our ability to adapt to the demands of our surroundings and, for example, to locate specific target items within a complex visual environment. In the visual search paradigm, participants are typically presented with an array of visual stimuli and are asked to identify and locate a predefined target within that array. However, the orienting of attention toward relevant items is usually not solely driven by salient perceptual information and concurrent, pre-specified task sets, but performance is also influenced by past experience, such as the learning of re-occurring, “statistical” regularities that the visual system registers in the environment.

For example, Chun and Jiang (1998) investigated whether the spatial configuration of items in a visual search display can guide attention. They argued that preserving the spatial arrangement of distractors across trials would allow participants to learn and use the arrangement to guide their attention more efficiently toward the target's location. They ran a series of experiments where they manipulated the target context. In the old context condition, the target was repeatedly presented within the same (old) arrangement of distractor items. In contrast, in the new context condition, the target was always presented within a new configuration of distractor items that was randomly generated on each trial. Blocks of 24 trials containing twelve old and twelve new configurations were presented repeatedly across the experiment. They showed that repeating the spatial context led to faster Reaction Times (RTs) than when the context was new. Chun and Jiang termed this RT effect Contextual Cueing. Furthermore, Chun and Jiang argued that learning the repeated context occurred incidentally, that is, without the participants' conscious effort to do so (but see also Smyth and Shanks, 2008).

The contextual cueing effect has been established in many studies over the last 25 years (for an overview, see Jiang and Sisk, 2019). The effect has been demonstrated across the lifespan (Merrill et al., 2013; but see also Kojouharova et al., 2023). Context learning also occurs using naturalistic scenes as stimuli (Brockmole and Henderson, 2006; Brockmole et al., 2006) and images of real-world objects (Makovski, 2016). The contextual cueing effect has been established in non-human primates, such as baboons (Goujon and Fagot, 2013) and pigeons (Wasserman et al., 2014). It occurs in children with ADHD (Weigard and Huang-Pollock, 2014), and even in dyslexic children who often show deficits in implicit sequence learning (Jiménez-Fernández et al., 2011). The acquisition of contextual memories (but not its adaptation) is enhanced in action video-game players (Zinchenko et al., 2022). The effect has been found under rapid stimulus presentation (Xie et al., 2020) and using a tactile search task, where learning occurs in an anatomical reference frame (Assumpção et al., 2018). An ongoing debate exists about whether contextual cueing depends on spatial working memory (Pollmann, 2019). For example, Travis et al. (2013) argued that spatial working memory is necessary to acquire contextual associations. In contrast, Manginelli et al. (2012, 2013) argue that working memory is only required to express learned associations. However, in a recent study, Vicente-Conesa et al. (2022) showed that contextual cueing was independent of working memory load.

**Figure 1:**
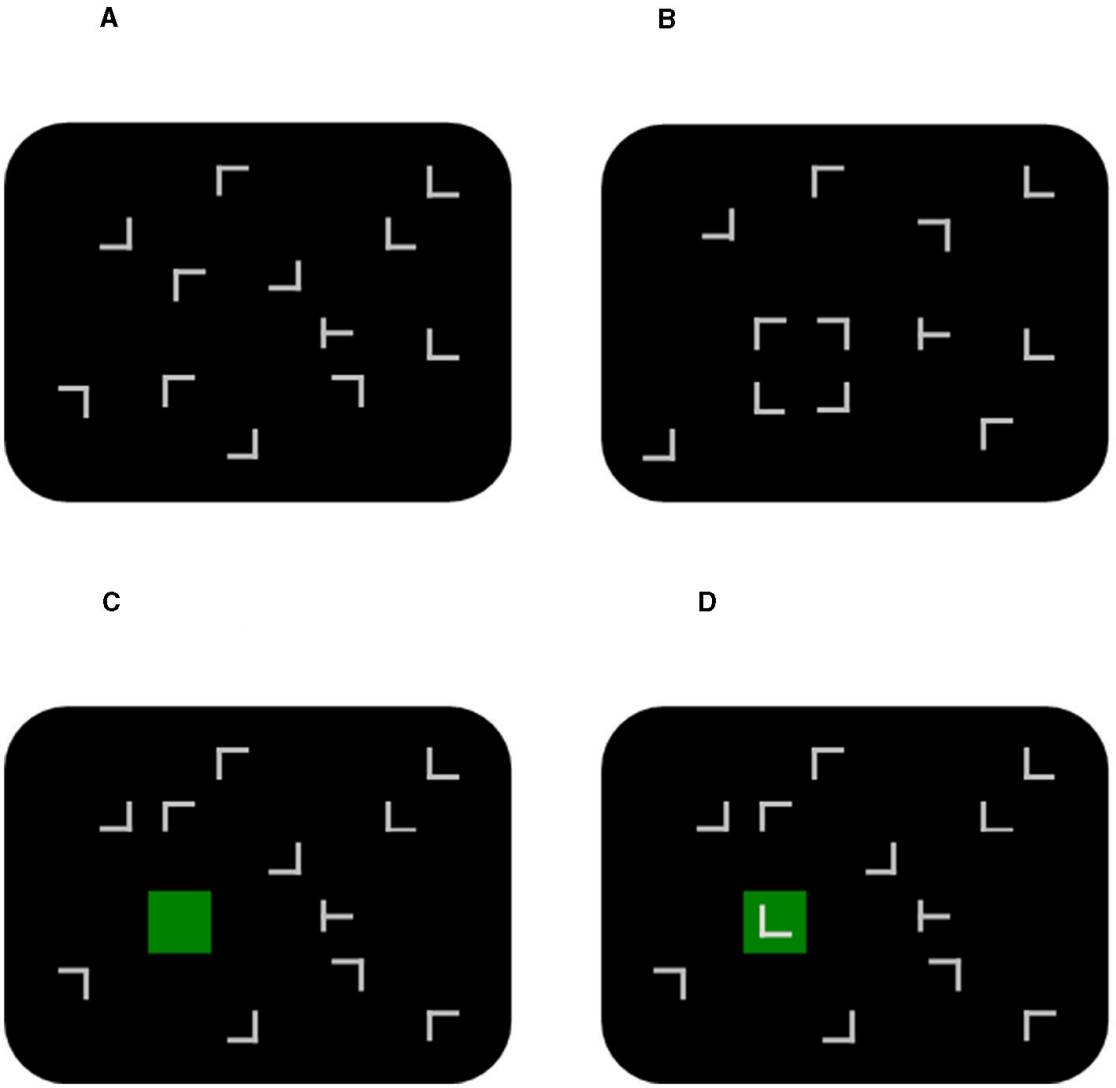
Examples of **(A)** a standard display (used in Experiments 1, 2, 4, 5), **(B)** a display with an illusory square formed by four non-target Ls [used by Conci and von Mühlenen (2009)], **(C)** a display with a non-overlapping green square (used in Experiments 1–5), and **(D)** a display with an overlapping green square (used in Experiments 2, 3). Note that stimuli are not drawn to scale.

Conci and von Mühlenen (2009) reported that the contextual cueing effect was reduced or even abolished if a salient feature or object was added to the search displays. For example, in one experiment, they used the typical T/L search task, and four distractor Ls were aligned to form an illusory square (see Figure 1B for an example). They predicted this illusory square would strengthen the contextual cueing effect by providing a more salient (predictive) cue for the target location. Instead, they found that contextual cueing did actually not occur at all with displays containing a square. They found the same result with displays containing an illusory cross instead of a square (i.e., the Ls forming the square were turned 180°). When the same four Ls did not form an illusory shape (i.e., the Ls were randomly oriented), the contextual cueing effect returned, but only half as strong as the standard contextual cueing effect (96 vs. 191 ms, respectively). Given this pattern of results, Conci and von Mühlenen argued that processing a figural grouping requires attention, which is diverted away from the overall context of the search display. As a result of this lack of attention to the repeating context, no (or only a reduced) RT facilitation was observed.

Kimchi and colleagues argued that the formation of such objects occurs automatically, capturing attention in a bottom-up manner (e.g., Kimchi et al., 2007; Yeshurun et al., 2009). Further evidence that this perceptual segmentation constrains contextual cueing such that the object automatically captures attention comes from Conci et al. (2013). They used the same type of displays, but the illusory squares were larger (i.e., 5 × 5 instead of 2 × 2 cells). They demonstrated that perceptual grouping affects contextual cueing such that targets presented within a segmented region benefit from contextual cueing, whereas targets outside the grouped region do not. This differential contextual cueing persisted even when the square was removed in the final epoch (transfer phase), suggesting that perceptual grouping primarily influences the learning of a given display layout (Conci and von Mühlenen, 2011).

In all these studies (Conci and von Mühlenen, 2009; Conci et al., 2013), the illusory shape was formed by elements that belonged to the search display (i.e., the four distractor Ls). Even though the target could never be among those four items, they might have kept some residual impact on the search process. Hence, being part of the display might be a critical factor for why the shape cannot be ignored and, consequently, why contextual cueing does not occur when the shape is present. This series of experiments uses the same basic paradigm as Conci and von Mühlenen (2009), but instead of an illusory square formed with Ls, we present an actual, filled square, which has little resemblance to the search stimuli. Suppose that the earlier commonalities between the square Ls and the distractor Ls were crucial for preventing contextual cueing. In that case, changing the square to a genuinely task-irrelevant object might reduce or abolish the square's impact on contextual cueing.

## Experiment 1

Experiment 1 was conducted to investigate whether adding a salient but task-irrelevant object affects the encoding of spatial context in visual search. We test whether adding a green square to the display (see Figure 1C) impedes memory-based attentional guidance to the target when using a contextual cueing paradigm (Chun and Jiang, 1998).

### Methods

#### Participants

Ten volunteers (2 male, 8 female, mean age 32.8 years, all right-handed) took part in the experiment. They received course credit or payment of 8€/h for their participation. All participants reported normal or corrected-to-normal vision. Each participant gave written informed consent before the experiment. The experimental procedure was in accordance with the Declaration of Helsinki and approved by the Ethics Committee of the Department Psychologie at Ludwig-Maximilians-Universität München. The sample size was based on Conci and von Mühlenen (2009), who reported robust effect sizes for this type of paradigm.

#### Apparatus, stimuli, and trial sequence

The participants were seated in a dimly lit, sound-proofed room in front of a 17” CRT monitor approximately 57 cm away. The monitor had a 1,024 × 786 pixels resolution and was controlled by an IBM-PC-compatible computer. The experiment was programmed in MATLAB using the Psychophysics Toolbox extensions (Brainard, 1997; Pelli, 1997; Kleiner et al., 2007). The Stimuli were drawn in gray (luminance 8.5 cd/m^2^) or green (luminance 6.9 cd/m^2^) against a black background (0.02 cd/m^2^). The letters and fixation cross had a width and height of 0.7° × 0.7° of visual angle, and the error feedback minus sign had a length of 0.7°, and they were all drawn at a thickness of one pixel (~0.13°). The filled green square was 2.5° × 2.5°. Responses were recorded using the right and left mouse buttons.

A search display consisted of twelve items, eleven non-targets and one target. Non-targets were L shapes randomly rotated in one of four orthogonal orientations. The target was a T shape randomly rotated 90° clockwise or counterclockwise. The letters were randomly placed within the cells of an invisible 8 × 6 matrix (cell size 2.5°). Additionally, their positions within each cell were randomly jittered (horizontally and vertically ±0.6° from the center of the cell) to avoid collinearities between letters. Two types of displays were generated: Standard displays had a fully randomized spatial layout (see Figure 1A for an example display). Square displays were like standard displays with the additional green square at a randomly selected unoccupied location in the matrix (see Figure 1C).

Each trial started with a central fixation cross for 0.5 sec, followed by the search display. The task was to search for the target and decide as quickly and accurately as possible whether the “stem” of the T was pointing to the left or right. Participants responded by pressing the corresponding button with their left or right index finger. After the response, the display was removed, and when the response was wrong, feedback was given by showing the error sign for 1 sec. The intertrial interval was 1 sec.

#### Design and procedure

The experiment had a within-subjects design with three independent variables: display type, context, and epoch. The first variable, display type, had two levels (standard or square), determining whether the green square was absent or present. The second variable, context, also had two levels (old or new). In the old-context condition, the arrangement of non-target items was the same (i.e., their locations and orientations) in every presentation. In the new-context condition, a new random arrangement of non-target items was generated on every presentation. The target appeared equally often at 24 possible locations throughout the experiment (i.e., 12 locations for old context and 12 different locations for new context) to rule out location probability effects. Whether the target was pointing left or right was determined randomly for each trial so that the old contexts were not predictive of the target orientation. Note that the location of the green square was also preserved in the old-context condition. The last variable, epoch, divided the experiment into six subsequent bins, allowing the assessment of possible learning effects over the course of the experiment.

Participants first completed a block of 24 randomly generated practice trials to get familiarized with the task, followed by 720 experimental trials subdivided into 30 blocks. Each block contained the same twelve old-context displays and twelve new-context displays, all presented in randomized order. Moreover, a block had twelve standard and twelve square displays; thus, there were six trials for each combination of display and context (standard/old, standard/new, square/old, square/new). An epoch consisted of five blocks, so the experiment had a total of 30 blocks grouped into six epochs. There were short mandatory breaks of 5 sec between blocks.

#### Recognition test

After the search task, participants were asked to perform a recognition test. They were informed that certain display configurations had been repeated throughout the experiment and that they had to indicate whether they recognized a given display arrangement. Of the 24 displays shown, 12 were the old-context displays used in the experiment, and 12 were newly generated. The trial sequence was identical to the search task, except that no feedback was given. Participants had to indicate whether they had previously seen the display or whether it was new. Non-speeded responses were recorded with the left mouse button for seen and the right button for new display arrangements.

### Results

#### Search errors

Mean error rates were calculated for each participant and variable combination. The overall error rate was very low (1.5%). A repeated measures ANOVA with the factors Display Type (standard, square), Context (old, new), and Epoch (1–6) revealed only a significant interaction effect between Display Type and Epoch, *F*_(5, 45)_ = 2.95, *p* = 0.022, $\eta_{\text{p}}^{2}$ = 0.247: this was due to error rates being slightly higher in the presence of a square; however, the effect occurred only in some epochs (on average 1.2% higher in epochs 1, 3, 4, and 6; on average 0.6% lower in epochs 2 and 5).

#### Search RTs

**Figure 2:**
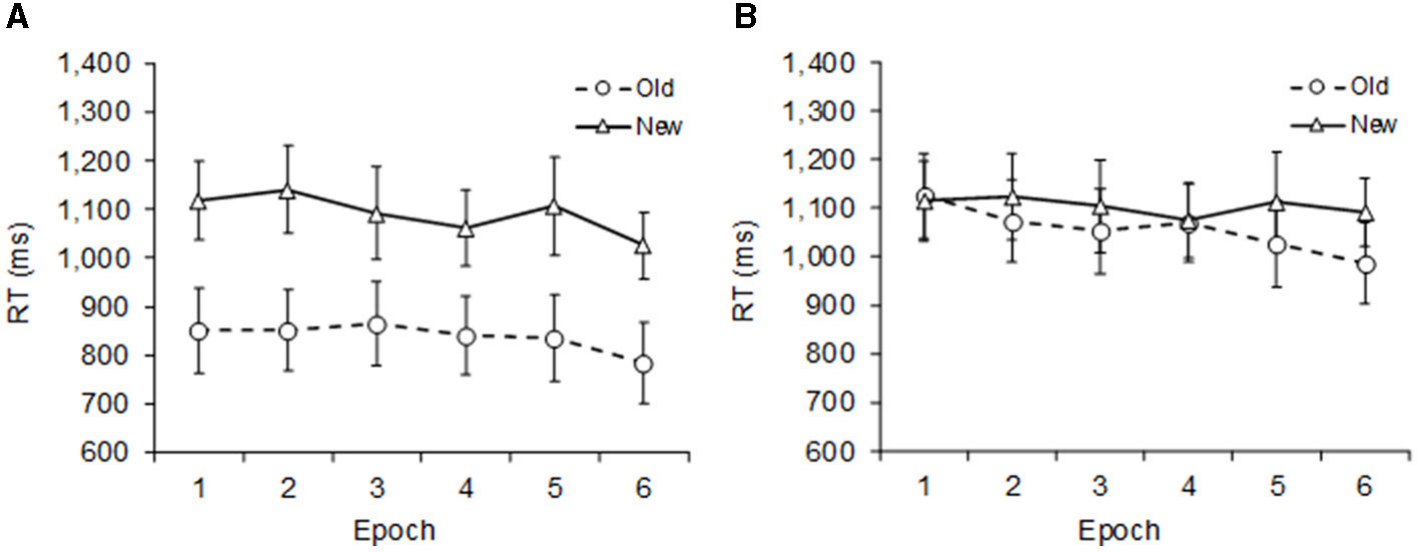
Mean correct RTs and SEM (error bars) in Experiment 1 as a function of epoch, with separate graphs for **(A)** standard displays and **(B)** square displays. Dashed lines represent old, and solid lines new context.

Mean correct RTs were calculated individually for each participant and each variable combination, excluding the erroneous responses and RTs >3 sec (< 1% of all trials, which was also the case in subsequent experiments). Mean correct RTs, averaged across participants, are shown in Figure 2 for standard displays (left panel) and square displays (right panel).

A repeated measures ANOVA with the factors Display Type (standard, square), Context (old, new), and Epoch (1–6) revealed significant main effects of Display Type, *F*_(1, 9)_ = 107.27, *p* < 0.001, $\eta_{\text{p}}^{2}$ = 0.922, Context, *F*_(1, 9)_ = 46.04, *p* < 0.001, $\eta_{\text{p}}^{2}$ = 0.836, and Epoch, *F*_(5, 45)_ = 3.84, *p* = 0.006, $\eta_{\text{p}}^{2}$ = 0.299: RTs were on average 115 ms faster in standard display than in square display trials, 150 ms faster in old-context than in new-context trials, and they became faster with increasing epoch (RTs decreased by 81 ms from Epoch 1 to 6). Furthermore, there was a significant interaction between Display Type and Context, *F*_(1, 9)_ = 24.20, *p* < 0.001, $\eta_{\text{p}}^{2}$ = 0.729, indicating that contextual cueing occurred more strongly with standard than with square displays (253 vs. 48 ms, respectively). Two split-up ANOVAs confirmed this interpretation: the Context main effect was significant in the standard display ANOVA, *F*_(1, 9)_ = 53.67, *p* < 0.001, $\eta_{\text{p}}^{2}$ = 0.856, but not in the square display ANOVA, *F*_(1, 9)_ = 3.45, *p* = 0.096, $\eta_{\text{p}}^{2}$ = 0.277. Furthermore, mean RTs were calculated separately for each block to explore the onset of contextual learning with standard displays. The contextual cueing effect emerged early, being significant already in Block 2, *t*_(9)_ = 3.22 *p* = 0.011, Cohen's d = 1.02. The subsequent experiments also revealed a similar pattern of rapid contextual cueing.

#### Recognition test

Overall, the mean accuracy in the recognition test was 45.9% with standard displays and 46.7% with square displays, *t*_(9)_ = 0.12, *p* = 0.904, Cohen's d = 0.039. The absence of a marked difference makes it unlikely that the contextual cueing effect with standard displays is due to better explicit memory for old contexts. However, this does not rule out the possibility that the repetition of old displays might have led to the formation of some explicit memory. To test this, one typically looks at whether the number of displays correctly recognized as old in the recognition test (hits) is larger than the number of new displays erroneously classified as old (false alarms). In order to increase the statistical power, we combined the recognition data of this and all subsequent experiments, which all used the same recognition procedure. The combined data of altogether 50 participants was analyzed with a 2 × 2 ANOVA with the factors Display Type (standard, square) and Response Type (hits, false alarms). There was a significant main effect for Response Type, *F*_(1, 49)_ = 12.00, *p* = 0.001, $\eta_{\text{p}}^{2}$ = 0.197, due to a higher hit rate compared to the false alarm rate (48.3 vs. 40.2%, respectively). This difference suggests that participants could identify at least some of the repeated displays, consistent with previous findings that tested larger samples (Vadillo et al., 2016; Geyer et al., 2020). Colagiuri and Livesey (2016) found in their sample (*N* = 766) that explicit memory was not associated with increased search facilitation, suggesting that the contextual cueing effect is independent of explicit recognition of the old context. Regarding Experiment 1, we would also like to add that the weak but above-chance explicit memory does not explain the observed reduction in contextual cueing with square displays because the (explicit) memory should have affected both display types to the same extent.

### Discussion

The results of Experiment 1 showed a robust contextual cueing effect for the standard displays: participants detected the target significantly faster within an old context than within a new context. This finding suggests that the memorized contextual information can be used to guide spatial attention to the target location, leading to an RT benefit for repeated displays (e.g., Chun and Jiang, 1998). The contextual cueing effect emerged rapidly (as early as block 2), a phenomenon that has also been reported in several other studies (e.g., Peterson and Kramer, 2001a; Schankin and Schubö, 2010; Makovski, 2016).

However, adding a task-irrelevant filled square to the display strongly impaired the contextual cueing effect. This finding seems remarkable because although the added square was irrelevant to the search task, its location would still have some predictive value regarding the target location in old-context displays. However, the finding aligns with Conci and von Mühlenen (2009), who found a similar effect using illusory squares formed by four Ls (see Figure 1B), which reduced contextual cueing from 231 with standard displays to −23 ms. As outlined in the introduction, it was expected that participants would be more inclined to ignore a genuinely task-irrelevant object, which, therefore, would not interfere with contextual learning.

Interestingly, when looking only at the new-context trials (cf. the solid lines in Figures 2A, B), RTs in the standard and square displays were very similar across all epochs (averaged across epochs: 1,055 vs. 1,065 ms, respectively, *p* > 0.653). The fact that search times were little affected by the squares may indicate that interference occurred more at the level of learning than at the level of searching the displays. It is worth noting that the brightness of the filled square was rather low and similar to the search stimuli (6.9 vs. 8.5 cd/m^2^, respectively). The square was, therefore, not very salient, which might thus explain why it did not capture attention (cf. Theeuwes, 1992).

Conci and von Mühlenen (2009) argued that figural grouping and attention interact with contextual cueing. However, it is unclear whether in their study the illusory square captured attention or not, because search times were, in fact, 86 ms faster in square displays, likely because the square reduced the number of items that needed to be inspected. In the current experiment, the square was clearly task-irrelevant, not very salient, and it did not influence the baseline search RTs. Yet, it still interfered with contextual cueing, which replicated these previous findings.

One possibility is that participants still see the green square as part of the display configuration because it fills a location that can thus not contain a search item. This indirect form of influence on the search display might have been sufficient to make it somehow relevant to the search process. Hence, its presence could require attentional resources that were then unavailable for processing and, consequently, learning the context in repeated displays. The square would not capture attention in a bottom-up manner, but it would prevent contextual learning by diverting attention away from the otherwise predictive context in repeated displays. This view would be in line with previous studies reporting that the implicit learning of contextual information critically depends on deploying selective attention to predictive information (Jiang and Chun, 2001; Jiang and Leung, 2005; Goujon et al., 2009).

## Experiment 2

It is possible that the square was seen as part of the search display in Experiment 1 because a search item could not occupy its location. Experiment 2 removes this location dependency by allowing the square to occupy any location, including distractor locations. In the case of an overlap, the distractor was presented superimposed on the square (see Figure 1D for an example display). The overlap gave the impression that the square appeared in a different depth plane further away from the search items. After some trials, this impression of a depth separation between the square and the search items might thus also generalize to the non-overlapping displays.

### Methods

Ten new volunteers (4 male, 6 female, mean age 29.8 years, all right-handed) took part in this study. All reported normal or corrected-to-normal vision and gave written informed consent. The apparatus, stimuli, and trial sequence were identical to Experiment 1. The only difference was that the green square could occur at any location in the display, including non-target locations (see Figure 1D). The letter was presented superimposed on the green square when their locations overlapped. The location of the green square was chosen randomly from all locations, excluding the target location. On average, the green square appeared in 11/47 (~23%) of all trials at an occupied (distractor) location. The design and procedure were the same as in Experiment 1.

### Results

#### Search errors

Erroneous responses were again rare (1.2%), and the ANOVA with the factors Display Type, Context and Epoch revealed no significant effects (all *p* > 0.114).

#### Search RTs

**Figure 3:**
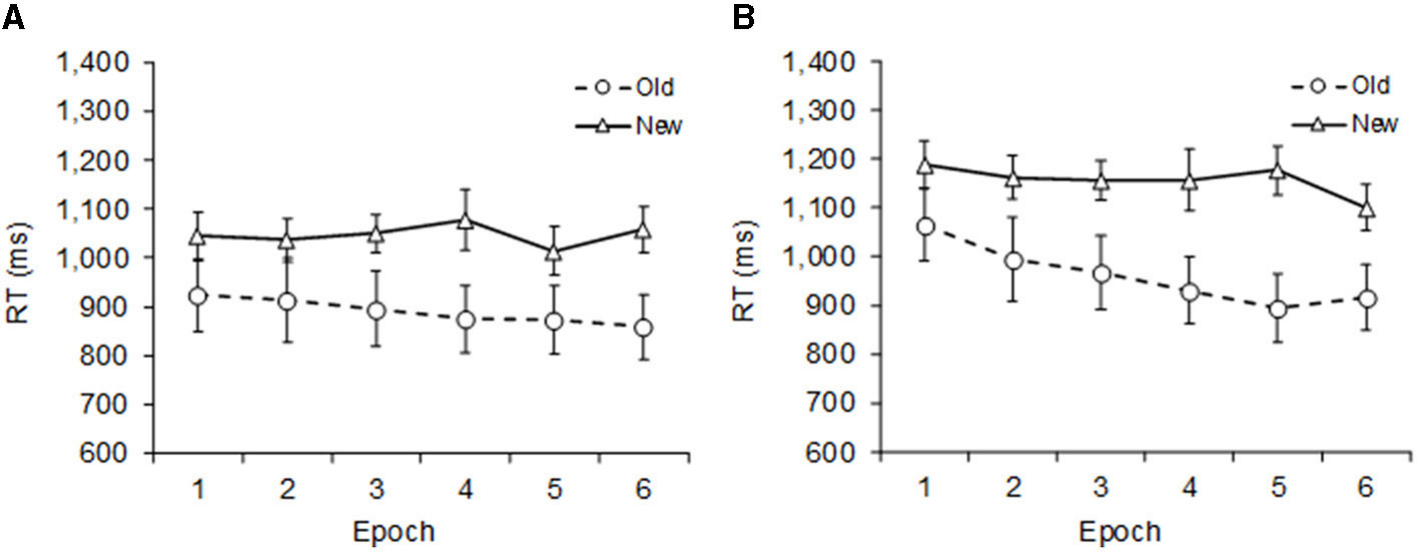
Mean correct RTs and SEM (error bars) in Experiment 2 as a function of epoch with separate graphs for **(A)** standard displays and **(B)** square displays. Dashed lines represent old, and solid lines new context.

Mean correct RTs for standard and square displays (excluding errors and outliers) averaged across participants are shown in Figure 3. A repeated measures ANOVA with the factors Display Type, Context, and Epoch revealed significant main effects of Display Type, *F*_(1, 9)_ = 16.02, *p* = 0.003, $\eta_{\text{p}}^{2}$ = 0.640, Context, *F*_(1, 9)_ = 17.78, *p* = 0.002, $\eta_{\text{p}}^{2}$ = 0.664, and Epoch, *F*_(5, 45)_ = 3.76, *p* = 0.006, $\eta_{\text{p}}^{2}$ = 0.294: RTs were, on average, 91 ms faster with standard than with square displays, 176 ms faster in old-context than in new-context trials, and they became 72 ms faster from epochs 1 to 6. There was a significant Display Type × Epoch interaction, *F*_(5, 45)_ = 2.69, *p* = 0.033, $\eta_{\text{p}}^{2}$ = 0.230, indicating that the RT advantage of standard over square displays steadily declines from 143 to 50 ms from epoch 1 to 6, respectively. Finally, there was a significant Context × Epoch interaction, *F*_(5, 45)_ = 3.49, *p* = 0.009, $\eta_{\text{p}}^{2}$ = 0.280, due to contextual cueing increasing from 123 to 191 ms from epoch 1 to 6, respectively. It is important to note that, in contrast to Experiment 1, there was no significant interaction involving Display Type × Context (2-way: *p* > 0.640, 3-way: *p* > 0.149), suggesting that the contextual cueing effect was equally strong with standard and square displays (157 and 196 ms, respectively).

#### Recognition test

Overall, the mean accuracy in the recognition test was 50.8% with standard displays, which was again statistically comparable to the recognition performance of 42.5% with square displays, *t*_(9)_ = 1.11, *p* = 0.296, Cohen's d = 0.351. For an analysis of hit vs. false alarm rates, see the overall analysis presented in Experiment 1.

### Discussion

The results of Experiment 2 showed a robust contextual cueing effect for *both* standard and square displays (it was actually 40 ms larger with square displays, but this was statistically not significant). In previous studies, adding an illusory square (Conci and von Mühlenen, 2009) or a green real square (Experiment 1) to the search display abolished or strongly reduced the contextual cueing effect. Compared to Experiment 1, it is astonishing that such a small difference, namely whether the square is allowed to overlap with search items or not, would have such a big effect on contextual cueing (196 vs. 48 ms, respectively, Experiment × Context, *F*_(1, 18)_ = 7.32, *p* = 0.014, $\eta_{\text{p}}^{2}$ = 0.289, in a corresponding ANOVA with square displays only).

Despite not interfering with context learning, overall search performance was slowed down by the presence of the square (when it was interpreted as appearing in the background). When looking at new-context trials (cf. the solid lines in Figures 3A, B), search times were significantly slower (160 ms) when the square was present, Display Type main effect, *F*_(1, 9)_ = 16.44, *p* = 0.003, $\eta_{\text{p}}^{2}$ = 0.646 in a corresponding new-context ANOVA. This finding is different from Experiment 1, where the square (appearing as part of the display) did not affect search times to a comparable extent (10 ms), at least in the first five epochs, Experiment × Display Type, *F*_(1, 18)_ = 2.91, *p* = 0.10, $\eta_{\text{p}}^{2}$ = 0.139, Experiment × Display Type × Epoch, *F*_(5, 90)_ = 2.51, *p* = 0.036, $\eta_{\text{p}}^{2}$ = 0.122, in a corresponding new-context ANOVA. One interpretation is that the search is slowed down by the square because it captures attention, thus drawing attention to the background plane. However, this finding is similar to Conci and von Mühlenen (2009, Experiment 2), where standard displays were compared with singleton displays, where the search array contained one red distractor. The red singleton did not affect the standard contextual cueing effect much, which remained relatively strong. However, in new-context trials, the overall search times were also significantly slower (106 ms) when the singleton was present. We will further consider these findings in the General Discussion.

## Experiment 3

Experiment 2 showed a strong contextual cueing effect when the square was allowed to overlap with the search items. This finding differs from Experiment 1, where the square never overlapped with the search items and where contextual cueing did not occur. We proposed that this difference might have to do with whether the square is seen as being part of the display (same depth plane) or not (different depth plane). Experiment 3 tests an alternative possibility: whether the contextual cueing effect in Experiment 2 arises only in the 23% of trials where the square would overlap with a distractor. For this purpose, Experiment 2 was repeated, but this time, displays always contained a green square (removing standard displays). In half the trials, the square was presented at an empty location and in the other half at an occupied distractor location. If contextual learning with squares in Experiment 2 only occurred in the overlapping trials, then Experiment 3 should allow us to see this difference between overlapping and non-overlapping squares in a better-controlled comparison.

### Methods

Ten new volunteers (5 male, 5 female, mean age 25.2 years, all right-handed) took part in this study. All reported normal or corrected-to-normal vision and gave written informed consent. The apparatus, stimuli, and trial sequence were identical to Experiment 2. The only difference was that the search displays would always contain a green square (i.e., there were no standard displays). The square would occur in half of the trials at an empty location and in the other half at an occupied distractor location (see Figures 1C, D for examples). The design and procedure were otherwise the same as in Experiment 2.

### Results

#### Search errors

The overall error rate was somewhat increased compared to the previous experiments (3.1%). An ANOVA with the factors Square Location (empty, occupied), Context (old, new), and Epoch (1–6) revealed a significant main effect of Context, *F*_(1, 9)_ = 10.40, *p* = 0.010, $\eta_{\text{p}}^{2}$ = 0.536, indicating a lower error rate with old than with new displays (2.5 vs. 3.6%, respectively).

#### Search RTs

**Figure 4:**
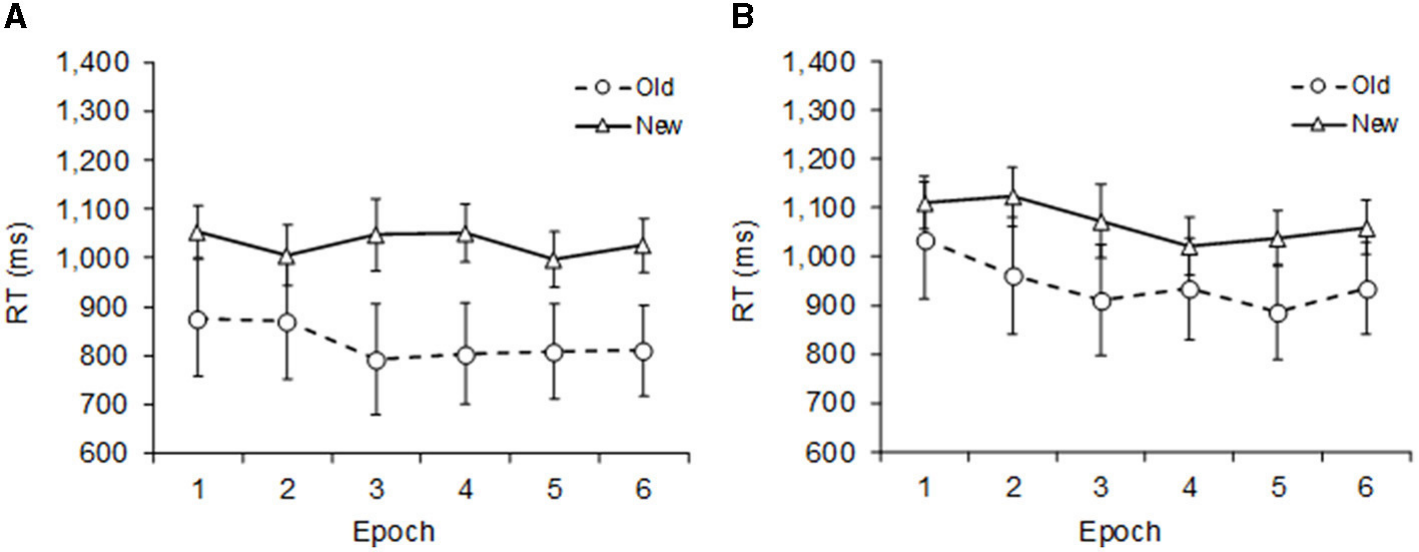
Mean correct RTs and SEM (error bars) in Experiment 3 as a function of epoch with separate graphs for when the square occurred **(A)** at an empty location and **(B)** at an occupied location. Dashed lines represent old, and solid lines new context.

Mean correct RTs for empty and occupied square locations (excluding errors and outliers) are shown in Figure 4. A repeated measures ANOVA with the factors Square Location, Context, and Epoch revealed significant main effects of Context, *F*_(1, 9)_ = 21.86, *p* = 0.001, $\eta_{\text{p}}^{2}$ = 0.708, and Epoch, *F*_(5, 45)_ = 5.40, *p* < 0.001, $\eta_{\text{p}}^{2}$ = 0.375. RTs were, on average, 165 ms faster in old-context than in new-context trials, and they became 63 ms faster from epoch 1 to 6. Furthermore, there was a significant 3-way interaction, *F*_(5, 45)_ = 3.31, *p* < 0.013, $\eta_{\text{p}}^{2}$ = 0.269: The contextual cueing effect was overall larger with empty than with occupied square locations (203 vs. 127 ms, respectively); however, this difference in contextual cueing was only significant in epoch 4, *t*_(9)_ = 2.74 *p* = 0.023, Cohen's d = 0.868 (247 vs. 87 ms, respectively), but not in the other epochs (all p's > 0.154, averaged cueing effects were 194 vs. 135 ms, respectively). The contextual cueing effect of 127 ms was in a separate ANOVA (only occupied square location) still significant, *F*_(1, 9)_ = 23.02, *p* < 0.001, $\eta_{\text{p}}^{2}$ = 0.719^1^.

Note that, even though RTs were slowed down by 79 ms when the square appeared at an occupied (compared to an empty) location, this effect did not reach statistical significance, Square Location main effect, *F*_(1, 9)_ = 3.20, *p* = 0.107, $\eta_{\text{p}}^{2}$ = 0.262. This slowing-down effect was even more negligible (41 ms) in new-context trials, Square Location main effect, *F*_(1, 9)_ = 2.25, *p* = 0.168, $\eta_{\text{p}}^{2}$ = 0.200, in a corresponding new-context ANOVA.

#### Recognition test

Overall, the mean accuracy in the recognition test was 43.4% with displays that presented the square at an empty location and 49.2% with displays that presented the square at an occupied location, *t*_(9)_ = 1.07, *p* = 0.312, Cohen's d = 0.339. For an analysis of hit vs. false alarm rates, see the overall analysis presented in Experiment 1.

### Discussion

A significant contextual cueing effect occurred irrespective of whether the square appeared at an empty or occupied location (203 and 127 ms, respectively). This finding refutes the proposal that contextual cueing only occurs when the square is presented at an occupied location. The contextual cueing effect was, in fact, numerically smaller when the square appeared at an occupied location (but this difference was statistically not significant). Hence, we can reject the idea that the square effect in Experiment 2 was solely driven by the overlapping displays.

The reliable cueing effect with non-overlapping displays in this experiment is also interesting in comparison to the findings with square displays in Experiment 1, which had the same type of non-overlapping displays, yet resulted in reliable contextual cueing (cf. Figure 4B vs. Figure 2B), Experiment × Context, *F*_(1, 18)_ = 4.77, *p* = 0.042, $\eta_{\text{p}}^{2}$ = 0.210. That is, the displays were identical in terms of their composition; the only difference was whether they were mixed with standard displays (Experiment 1) or overlapping square displays (Experiment 3). It seems that this context of being mixed is the key factor determining whether contextual cueing is turned off or on. We believe these other trials in a block were defining whether the squares were seen as part of the search display (Experiment 1) or as part of a different depth plane in the background (Experiments 2, 3).

In new-context trials (cf. the solid line in Figures 4A, B), overall search times were somewhat slowed numerically, though not significantly by 41 ms when the square overlapped with a distractor location. This lack of a reliable difference suggests it is unlikely that the larger (160 ms) slow-down effect when the square was present in Experiment 2 is due to some visual interference occurring in the overlapping displays. It may, however, indicate that the less frequent appearance of the square in Experiment 2 (50% of all trials, compared to 100% in Experiment 3) resulted in a stronger attentional capture effect. This finding also suggests that contextual learning in the presence of a square in Experiments 2 and 3 happens across all trials, independent of whether the square overlapped with a distractor location or not.

## Experiment 4

The next two experiments further investigate the origins of the findings in Experiment 1, namely whether the absence of contextual cueing was because the added square prevented the acquisition of configural associations (e.g., Travis et al., 2013), or because it prevented the expression of learned associations (e.g., Jiang and Leung, 2005; Goujon et al., 2009; Conci and von Mühlenen, 2011; Manginelli et al., 2012). This was tested by changing the display composition in the last epoch. In Experiment 4, the learning phase (epochs 1–5) was identical to the corresponding epochs in Experiment 1. In the transfer phase (epoch 6), the square was then removed from the display. If the presence of a square hinders the manifestation of contextual cueing (and not the learning *per se*), then contextual cueing should return once the square is removed.

### Methods

Ten new volunteers (4 male, 6 female, mean age 23.8 years, all right-handed) took part in this study. All reported normal or corrected-to-normal vision and gave written informed consent. The apparatus, stimuli, and trial sequence were identical to Experiment 1 (i.e., the square and the letters never overlapped; see Figure 1C). The only difference was the addition of a transfer phase occurring in epoch 6, where the green square was removed from all square displays while the context (i.e., the configuration of the letters) remained unchanged (see Figure 1A). The design and procedure were the same as in Experiment 1^2^.

### Results

#### Search errors

The overall error rate (2.2%) was low. An ANOVA with the factors Display Type (standard, square), Context (old, new), and Epoch (1–6) revealed no significant effects (all *p*'s > 0.050).

#### Search RTs

**Figure 5:**
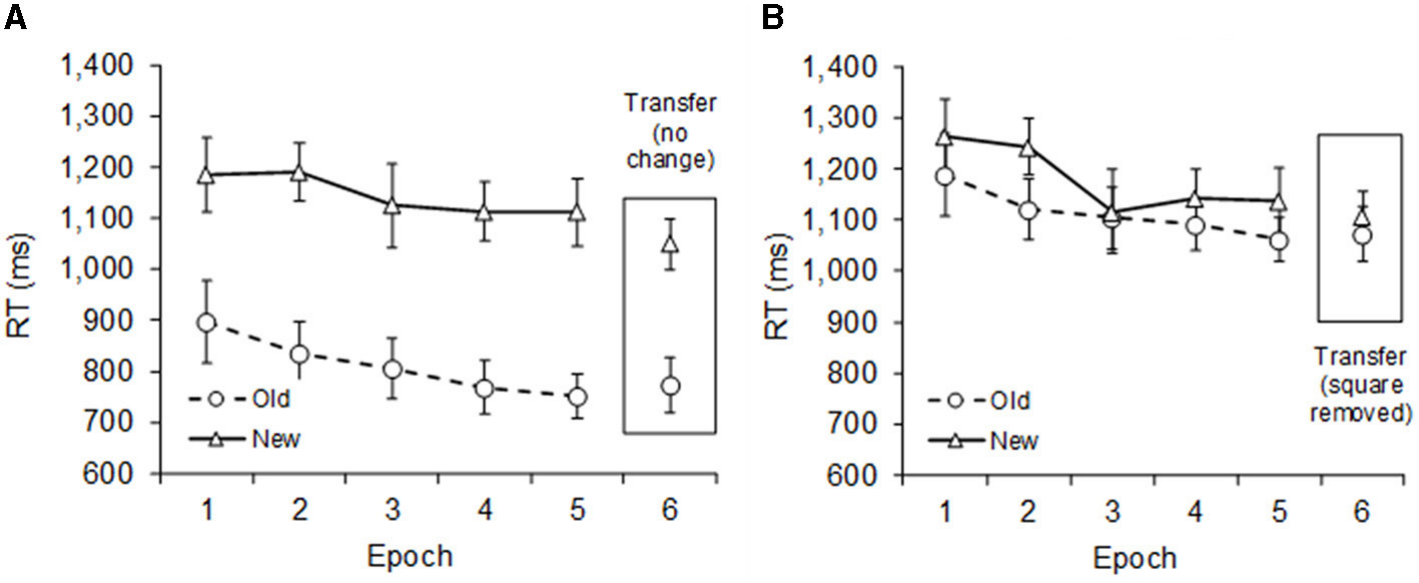
Mean correct RTs in Experiment 4 as a function of epoch with separate graphs for **(A)** standard displays and **(B)** square displays. During the transfer phase (epoch 6), the square was removed from the square displays (thus, essentially presenting “standard” displays). Dashed lines represent old, and solid lines represent new context.

Mean correct RTs for standard and square displays (excluding errors and outliers) are shown in Figure 5. The learning-phase data (epoch 1–5) was subjected to a repeated measures ANOVA with the factors Display Type (standard, square), Context (old, new), and Epoch (1–5). There were significant main effects of Display Type, *F*_(1, 9)_ = 61.21, *p* < 0.001, $\eta_{\text{p}}^{2}$ = 0.872, Context, *F*_(1, 9)_ = 152.75, *p* < 0.001, $\eta_{\text{p}}^{2}$ = 0.944, and Epoch, *F*_(4, 36)_ = 13.33, *p* < 0.001, $\eta_{\text{p}}^{2}$ = 0.597: RTs were on average 169 ms faster with standard than with square displays, 200 ms faster in old-context than in new-context trials, and they became 119 ms faster from epoch 1 to 5. As in Experiment 1, there was also a significant Display Type × Context interaction, *F*_(1, 9)_ = 31.37, *p* < 0.001, $\eta_{\text{p}}^{2}$ = 0.777, indicating that the contextual cueing effect was larger with standard than with square displays (333 vs. 67 ms, respectively). Two further split-up ANOVAs showed that the Context main effect was significant in both the standard-display ANOVA, *F*_(1, 9)_ = 78.14, *p* < 0.001, $\eta_{\text{p}}^{2}$ = 0.897, and the square-display ANOVA, *F*_(1, 9)_ = 19.72, *p* = 0.002, $\eta_{\text{p}}^{2}$ = 0.687. However, the significant interaction shows that the contextual cueing effect was, compared to standard displays, significantly reduced by the presence of a square.

The transfer-phase data (epoch 6) was subjected to an additional ANOVA with the factors Display Type and Context. It revealed significant main effects of Display Type, *F*_(1, 9)_ = 56.06, *p* < 0.001, $\eta_{\text{p}}^{2}$ = 0.862, and Context, *F*_(1, 9)_ = 57.50, *p* < 0.001, $\eta_{\text{p}}^{2}$ = 0.865, and a significant Display Type × Context interaction, *F*_(1, 9)_ = 21.67, *p* = 0.001, $\eta_{\text{p}}^{2}$ < 0.707. Contextual cueing continued to be as strong as in previous epochs with the standard displays; however, contextual cueing did not emerge when the square was removed from the square displays (277 vs. 34 ms, respectively). *Post-hoc t*-tests revealed that the contextual cueing effect was only significant with standard displays, *t*_(9)_ = 7.55 *p* < 0.001, Cohen's d = 2.386, but not with square displays, *t*_(9)_ = 1.15 *p* = 0.279, Cohen's d = 0.365.

#### Recognition test

Overall, the mean accuracy in the recognition test was 42.5% with standard displays and 49.2% with square displays, *t*_(9)_ = 1.16, *p* = 0.276, Cohen's d = 0.367. As for Experiment 1, we would like to add that the weak but above-chance explicit memory does thus not explain the observed reduction in contextual cueing with square displays because the memory should have affected both display types to the same extent (if anything, memory was, in fact, better for square displays). For an analysis of hit vs. false alarm rates, see the overall analysis presented in Experiment 1.

### Discussion

The pattern of results in the learning phase (epoch 1–5) of this experiment replicates the findings in Experiment 1. Compared to standard displays, the contextual cueing effect was reduced by 80% in the presence of a square, which is similar to the 81% reduction found in Experiment 1. When looking only at the new-context trials (see Figure 5), RTs differed little between standard and square displays, with an average difference of 39 ms across epochs 1–5 (*p* = 0.123). This difference is again similar to Experiment 1, where the difference was 13 ms across epochs 1–6. For both experiments, this suggests that the square did not interfere much with the search process; nonetheless, the square drastically reduced the contextual cueing effect.

In the transfer phase (epoch 6), removing the square from the display revealed no evidence of hidden learning. The contextual cueing effect in epoch 6 was not significant, and it was even smaller than in the preceding learning phase (34 vs. 67 ms, respectively). This lack of an effect is in stark contrast to the standard displays, where the contextual cueing effect in epoch 6 continued to be as strong as in the learning phase (277 vs. 333 ms, respectively). Overall, these results suggest that the presence of a square hinders learning because the predicted sudden manifestation of contextual cueing did not occur after the square was removed. It is also interesting to note that despite removing the square in epoch 6, there was no evidence for rapid learning of old contexts, which fits with previous studies showing that the learning of a new set of old contexts is possible but takes more time (e.g., Zellin et al., 2013).

## Experiment 5

The findings in the previous experiment suggest that the presence of a square prevented the learning of old displays in Experiment 1 and 4. The final experiment tests whether adding a square during the transfer phase prevents the manifestation of contextual cueing built up with standard displays during the learning phase.

### Methods

Ten new volunteers (6 male, 4 female, mean age 25.4 years, nine right-handed) took part in this study. All reported normal or corrected-to-normal vision and gave written informed consent. The apparatus, stimuli, and trial sequence were similar to Experiment 4. The main difference was that green squares were absent during the learning phase (epoch 1–5) but added to half of the displays during the transfer phase (epoch 6). Again, the context (i.e., the configuration of the letters) did not change, but only the square was added to an empty display location during transfer. The design and procedure were the same as in Experiment 1.

### Results

#### Search task

##### Search errors

The overall error rate was comparable to the previous experiments (2.0%). An ANOVA with the factors Display Type (standard, square), Context (old, new), and Epoch (1–6) revealed no significant effects (all *p*'s > 0.170).

##### Search RTs

**Figure 6:**
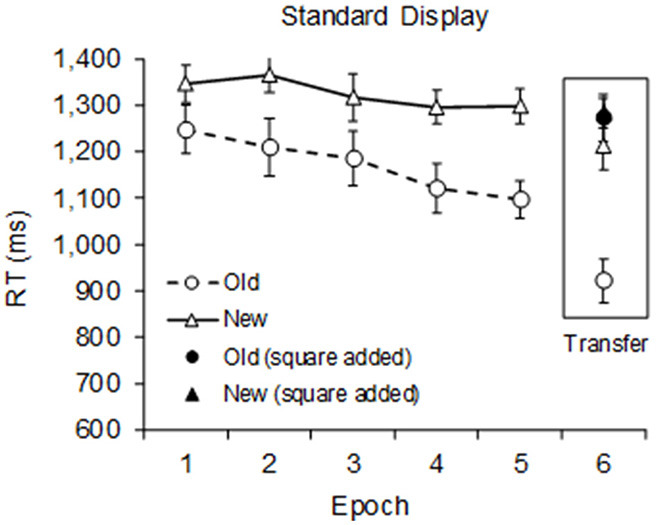
Mean correct RTs in Experiment 5 as a function of epoch. Only standard displays were used during the learning phase (epoch 1–5). During the transfer phase (epoch 6), a square was added to half of the displays. Circles represent old-context and triangles new-context trials. Note that in epoch 6, the “New (square added)” symbol is occluded by the “Old (square added)” symbol.

Mean correct RTs for standard displays (excluding errors and outliers) are shown in Figure 6. The learning-phase data was subjected to a repeated measures ANOVA with the factors Context (old, new) and Epoch (1–5). The results showed significant main effects of Context, *F*_(1, 9)_ = 45.32, *p* < 0.001, $\eta_{\text{p}}^{2}$ = 0.834, and Epoch, *F*_(4, 36)_ = 3.72, *p* = 0.012, $\eta_{\text{p}}^{2}$ = 0.293. RTs were, on average, 152 ms faster in old-context than in new-context trials, and they became 99 ms faster from epoch 1 to 5.

The ANOVA on the transfer phase data (epoch 6) with the factors Display Type (standard, square) and Context (old, new) showed significant main effects of Display Type, *F*_(1, 9)_ = 61.63, *p* < 0.001, $\eta_{\text{p}}^{2}$ = 0.873, and Context, *F*_(1, 9)_ = 30.02, *p* < 0.001, $\eta_{\text{p}}^{2}$ = 0.769, and a significant Display Type × Context interaction, *F*_(1, 9)_ = 9.48, *p* = 0.013, $\eta_{\text{p}}^{2}$ < 0.513: The contextual cueing effect continued to be strong with standard displays, but was almost entirely abolished by the addition of a square (289 vs. 11 ms, respectively). *Post-hoc t-*tests confirmed this interpretation, with a significant contextual cueing effect with standard displays, *t*_(9)_ = 5.13 *p* < 0.001, Cohen's d = 1.622, but not with square displays, *t*_(9)_ = 0.22 *p* = 0.825, Cohen's d = 0.072.

##### Recognition test

Overall, the mean accuracy in the recognition test was 41.8% with standard displays and 40.8% with square displays, *t*_(9)_ = 0.20, *p* = 0.843, Cohen's d = 0.064. For an analysis of hit vs. false alarm rates, see the overall analysis presented in Experiment 1.

### Discussion

The results in the learning phase with standard displays show a robust contextual cueing effect of 152 ms, comparable to the ones found in the previous experiments. During the transfer phase, a square was added to half of the displays (old and new), which abolished the contextual cueing effect (11 ms). Again, this lack of effect is in stark contrast to the other half of unchanged displays (i.e., standard displays), where the contextual cueing effect continued to be very strong (289 ms). We can only speculate whether the added square changes the overall representation of the learned configuration or whether it simply interferes with the retrieval of the learned information (e.g., Pollmann, 2019).

Overall, these results suggest that the presence of a non-overlapping square does not only hinder the acquisition, but also the manifestation of (previously) learned contextual information. It is also worth noting that the results in the transfer phase represent another replication of the findings in Experiments 1 and 4, which showed that the presence of a non-overlapping square can remove (or lessen) the contextual cueing effect.

## General discussion

The present set of experiments investigated how presenting a task-irrelevant object would interact with learning the configural information in the contextual cueing paradigm. In all experiments, very robust contextual-cueing effects (150–300 ms) were obtained with the standard displays. However, when a task-irrelevant square was added to the display, the contextual cueing effect was lessened or diminished (10–70 ms). These findings thus replicate the results of Conci and von Mühlenen (2009), who presented illusory squares formed by non-target items. However, when the added square was allowed to overlap occasionally with a search item in Experiments 2 and 3, contextual cueing emerged again. This “re-emerged” contextual cueing effect occurred irrespective of whether the square actually overlapped with an item or not, though the effect was more substantial when they overlapped (see Experiment 3). We proposed that the occasional overlap led to the impression of the square appearing in a different depth plane further away from the search items, where it would not interfere with contextual cueing. Finally, the last two experiments replicated the findings of Experiment 1 with non-overlapping displays. They also showed that the added square does not only hinder the acquisition but also the manifestation of learned contextual information.

Taken together, these experiments show that the presence of a task-irrelevant object can have a strong influence on memory-based contextual associations. However, these objects did not disrupt contextual cueing in all instances, only when it was seen as part of the display. When the object was seen as belonging to the background, contextual cueing returned. We believe that this can be explained with a bottom-up display segmentation process, which interacts with the learning of contextual information. When the square is grouped with the search items because it appears in the same plane, then the square attracts attention, which is diverted away from the overall context of the search display.

Another puzzling finding is that when the square has a distracting effect on contextual cueing (Experiments 1, 4, and 5), there was no (or only little) difference in overall RT. Such an RT difference has typically been taken as an indicator of attentional capture (Theeuwes, 1992). On the other hand, when the square had no such distracting effect on contextual cueing (Experiment 2), there were marked RT costs of around 110 ms in the new-context trials for when a square was presented (a summary of this relationship is presented in Table 1). This is similar to Peterson and Kramer (2001a,b), who combined contextual cueing with an abrupt visual onset paradigm, where a new distractor was added to the display. Such abrupt onsets typically show strong effects on attention (Jonides and Yantis, 1988). Peterson and Kramer found that the onset delayed RTs (indicating attention capture), but it did not affect contextual cueing (i.e., onsets affected both old and new configurations in the same way). They argued that onset distractors and repeated context had independent and opposing influences on search efficiency (Peterson and Kramer, 2001b). Similarly, Conci and von Mühlenen (2009) showed that the presence of a red singleton in the display would delay RTs but have little effect on contextual cueing.

**Table 1:** Summary of effects for same or different depth planes of square and search items.

**Experiments**	**Depth plane**	**RT costs**	**Contextual cueing**
1, 4, and 5	Same	No	No
2	Different	Yes	Yes

RT costs for squares could not be calculated in Experiment 3 because there was no standard display condition to compare with.

The attribution of the square as belonging to the foreground or background can only be reached after several trials (when the square happened to overlap with search items or not), and this could, thus, be considered a “global” regularity. In other words, not only the arrangement of the search display itself plays a role, but contextual cueing can also be influenced by “global” factors (i.e., via variations that occur across trials; see Jungé et al., 2007; Tseng et al., 2011; Zinchenko et al., 2018, 2023).

Zang et al. (2016) examined the impact of foreground-background segmentation on contextual cueing. They overlaid the search items with a task-irrelevant cuboid shape, which segmented the display into foreground and background. Results showed that even though the cuboid itself was not predictive of the target location, it was encoded in the representation, driving the contextual cueing effect. When the cuboid (which was constant during the learning phase) was rotated by 90° or entirely removed in the subsequent transfer phase, the contextual cueing effects were diminished. They concluded that foreground-background segmentation occurred prior to contextual learning, and only objects or arrangements that were grouped as foreground were learned over the course of trials.

These results are also supported by Conci et al. (2013), who argued that segmentation provides a basic structure within which contextual scene regularities are acquired. They used illusory squares that were made from four Ls that comprised a larger region of the search display, subtending 5 × 5 matrix locations. Using these types of displays, they showed that contextual cueing only occurred when targets were located within the region of the square (and not when they were outside the region). They concluded that contextual learning might be constrained by object-based selection. It thus seems that the region of the square became segregated from the rest of the display, with the segregated object region being prioritized during learning over the other display locations that were assigned to the background.

In another study, Zang et al. (2017) showed that contextual cueing under 3D viewing conditions is primarily based on 2D inter-item associations. In their experiment, stimuli were presented via a 3D-compatible projector, and participants wore a pair of 3D shutter glasses, allowing to present different images to each eye. They concluded that depth-defined spatial regularities are probably not encoded during contextual learning (see also Beesley et al., 2022, for a comparable finding). For a similar finding with displays that were segmented by means of color or size, see Conci and von Mühlenen (2011). This finding contrasts with Kawahara (2003), where contextual cueing was reduced when the disparity of distractors but not that of the target was reversed. However, Zang et al. (2017) argued that in Kawahara's study, the spatial configuration of the target's neighboring items changed to a different depth plane, potentially weakening binocular disparity variations. Thus, the reduction of contextual cueing might have resulted from changes to the learned inter-item associations within particular depth planes and not because of pure binocular disparity-defined depth variations.

In sum, the present study suggests that depth segregation modulates whether a task-irrelevant distractor interferes with contextual cueing or not. When the distractor is seen as part of the search display, then efficient learning of the display context is prevented.

## Data availability statement

All data for the reported experiments are publicly available via the Open Science Framework and can be accessed at https://osf.io/sy2ea/.

## Ethics statement

The studies involving humans were approved by Ethics Committee of the Department Psychologie at Ludwig-MaximiliansUniversität München. The studies were conducted in accordance with the local legislation and institutional requirements. The participants provided their written informed consent to participate in this study.

## Author contributions

AM: Conceptualization, Funding acquisition, Project administration, Validation, Writing – original draft. MC: Conceptualization, Data curation, Formal analysis, Methodology, Software, Writing – review & editing.

## Footnotes

^1^We also tested for order effects, breaking down the data depending on whether the square location in the previous trial was empty or occupied. The analysis showed that at empty locations, the contextual cueing effect was somewhat smaller (19%) when the previous square location was occupied compared to when it was empty (188 vs. 233 ms, respectively), though this difference was statistically not significant, *p* > 0.64. Moreover, at occupied locations, the contextual cueing effect did not differ (128 vs 129 ms, respectively). This suggests that contextual cueing produced a constant benefit, irrespective of whether the square location was overlapping in the previous trial or not.

^2^Note that there were effectively only standard displays in the transfer phase, but we continued to use the label “square display” to refer to the displays that had a square during the learning phase.
